# Characteristics of non-fatal overdoses and associated risk factors in patients attending a specialist community-based substance misuse service

**DOI:** 10.1177/20494637221095447

**Published:** 2022-05-24

**Authors:** Riya Ghose, Fiona Cowden, Abirami Veluchamy, Blair H Smith, Lesley A Colvin

**Affiliations:** 1 Department of Medicine, School of Medicine, University of Dundee, Dundee, UK; 2 Tayside Substance Misuse Service, Dundee, UK; 3 Department of Population Health and Genomics, School of Medicine, University of Dundee, Dundee, UK; 4 Department of Population Health Sciences, Medical Research Institute, Dundee, UK

**Keywords:** Opioids, benzodiazepines, gabapentinoids, methadone, buprenorphine, opioid replacement therapy, non-fatal overdose, substance use, drug-related deaths

## Abstract

**Introduction:**

There are concerns about rising drug-related deaths and the potential contribution of prescription analgesics. There is limited understanding regarding the role of prescription analgesics in non-fatal overdoses (NFODs), nor is there a good understanding of what factors are associated with more severe overdose.

**Objectives:**

To explore risk factors and characteristics of NFODs among people attending a specialist community-based substance misuse service.

**Methods:**

After Caldicott approval, data on NFODs, in people attending the Tayside Substance Misuse Service (TSMS), were extracted from the Scottish Ambulance Service database, along with opioid replacement therapy (ORT) prescribing data. Statistical analysis was performed using R studio and Microsoft Excel.

**Results:**

557 people (78% [434/556] male, mean age ± standard deviation 38.4 ± 7.95) had an NFOD. Repeat NFODs were more likely in males compared to females (*p* < .0065). Males were more likely to be administered naloxone (OR = 1.94, 95% CI = 1.10–3.40, *p* < .02). NFODs at home were more likely to be moderate to severe (categorized by Glasgow Comma Scale [*p* < .02, OR = 4.95, 95% CI = 1.24–24.38]). Methadone (321/557, 57.63%), benzodiazepines (281/557, 50.45%) and heroin (244/557, 43.81%) were the commonest substances: prescribed methadone overdose was more likely than buprenorphine (*p* < .00001). Opioids and benzodiazepines were often taken together (275/557, 49.40%), with almost all gabapentinoid NFODs also involving opioids (60/61, 98.40%).

**Conclusions:**

Polysubstance use with opioids prescribed for ORT, such as methadone, is highly likely to be implicated in NFOD, with males being at the highest risk of severe and repeat NFOD. Future work should focus on strategies to further reduce NFODs.

## Introduction

The continued increase in drug deaths globally, and more particularly in Scotland, with the highest recorded rate in Europe, poses a public health concern.^1^ Drug deaths often involve polypharmacy and can be caused by illicit drugs or legally obtained prescription drugs, with increasing concerns around the role of prescription analgesics.^2^

Factors that have been shown to cause a higher risk of opioid overdose include the following: having a current problem or history of substance use, taking prescribed medications (such as benzodiazepines) that interact with opioids, taking high-doses of opioids for a long period of time, being in receipt of a methadone prescription and having mental health or cognitive impairment issues.^3^ In the United States, as many as 5–7 million patients with substance misuse and/or addiction also suffer from pain, with the incidence having been found to be considerably greater, than the general population, in people on Methadone Maintenance Treatment.^4^ Chronic pain is common, affecting between one-third and one-half of the UK population, with around 14% having moderate to severe pain and disability.^5^ Any increases in analgesic prescribing may increase the potential for misuse and drug diversion. The significant increase in opioid prescribing has been well documented globally, and also within Scotland.^6,7^ Gabapentinoids, used in neuropathic pain management, have also seen an increase in prescribing in recent years, with potential for misuse.^8,9^ National and international guidelines recommend gabapentin and pregabalin as first-line treatment for neuropathic pain.^8,10^ Even though opioids and gabapentinoids can be effective in treating pain, they may be subject to diversion, and result in addiction, with harmful central nervous system (CNS) effects, particularly when taken together.^11^ Benzodiazepines are hypnotics and anxiolytics for short-term treatment of severe anxiety.^12^ Long-term use is not recommended, as this can lead to dependence, especially in patients with history of drug and alcohol use and personality disorder.^12^ Benzodiazepine prescribing has reduced in primary care in Scotland due to concerns about dependence and inappropriate prescribing, but the illicit market supply of benzodiazepines has risen.^13^

The proportion of drug-related deaths (DRDs) caused by a combination of opioids and gabapentinoids in Scotland rose from 0% in 2009 to 23% in 2016.^9,14^ According to the Drug-related Deaths in Scotland 2020 report, 93% of all DRDs involved more than one substance.^15^ Opioids (including heroin, morphine and methadone) and ‘street’ benzodiazepines (etizolam) were the most prevalent drugs, being implicated in 89% and 73% of the DRDs, respectively.^15^ From the Drug Deaths in Tayside 2019 report, 49/89 (55%) individuals who died, had experienced a non-fatal overdose (NFOD) previously.^16^ NFODs are therefore a risk indicator for DRDs and better understanding of NFODs might allow early-targeted intervention and reduction in DRDs.^16,17^ Around half (44/89, 49%) of people experiencing DRDs were engaging with a specialist substance misuse service (SSMS) at the time of death. Opioid replacement therapy (ORT), using controlled amounts of longer acting but less euphoric opioids (methadone or buprenorphine), is the standard approach used by SSMS to treat opioid dependence, aiming to reduce cravings and illicit substance use, as well as preventing withdrawal symptoms.^18^ The aim of this study was to understand the characteristics of NFODs in people attending an SSMS and to identify the risk factors associated with repeat and/or more severe NFODs, to inform service improvement work.

## Methods

### Data sources

The regional ambulance service regularly provides the regionalNational Health Service (NHS) SSMS with information about patients under their care, who have been treated by the ambulance service for an NFOD. Data from this source, on people who had an NFOD between 05/12/2017 and 12/05/2019, were analyzed. The information in the dataset included basic demographics such as the community health index (CHI) number (a unique identifier used for any person who has an NHS Scotland interaction), name, gender, date of birth, address and postcode, as well as details about the incident, including time and site of overdose, drugs consumed, use of naloxone, Glasgow Coma Scale (GCS), involvement of police and number of previous NFODs. The drugs consumed were self-reported by patients, as routine toxicology testing is not currently done by the ambulance service personnel. Reported use of more than one drug was considered to be polydrug use. Postcode was used to record the Scottish Index of Multiple Deprivation (SIMD) quintile for each individual’s home address. The SIMD is a standard approach to identify areas of deprivation in Scotland by ranking 6976 areas from those in the most deprived quintile (ranked 1) to those in the least deprived (ranked 5), based on income, employment, education, health, access to services, crime and housing.^19,20^ The CHI number was used to obtain details of ORT being prescribed by the SSMS. The regional SSMS provides recovery-orientated services for drug and alcohol use, using a multi-disciplinary approach, with community and inpatient programmes, with prescribed treatments for opioid and alcohol use.

### Data processing and analysis

Microsoft Excel (2013) and R studio (v1.2) were used for data processing and analysis.^21,22^ Descriptive statistics were used to understand the demographics of the dataset. Independent sample t-test was used to explore the effect of gender on NFOD. Chi-square testing was used to explore the relationship between NFOD and ORT prescription. Multi-variate logistic regression was used to assess factors associated with moderate to severe NFODs and we calculated the odds ratios (OR) and confidence interval (CI) using generalized linear models in R. The factors associated with the severity of NFODs were assessed using three models: the Glasgow Coma Scale (GCS), the administration of naloxone and the effects of reported single or polydrug use. Naloxone is an opioid antagonist that can be used to reverse the effects of opioid overdose, such as airway loss and respiratory depression.^23^ The ambulance service personnel used GCS to record the level of consciousness of the people who had experienced NFODs. Eye response, verbal response and motor response are measured to give patient a score between 1 and 15. A score below 8, between 9 and 12, 13 and above is classified as consciousness level being severely, moderately and mildly affected, respectively.^24^

### Approvals

Caldicott approval (SMED Caldicott Guardian Approval 19/25) was obtained from the regional NHS Caldicott Guardian. After discussion with the University of Dundee School of Medicine Ethics Committee, it was clarified additional approvals were not required.

## Results

The ambulance service dataset comprised 557 patients who had experienced an NFOD between 05/12/2017 and 12/05/2019. ORT prescription data from SSMS were not available for 118 patients, thus giving ORT data on 439 of 557 patients with a reported NFOD.

Table 1 shows the demographics of people who were reported to have had an NFOD by the ambulance service. The mean age (±standard deviation; range) was 38.40 (±7.95; 18–63) years. The majority of people (303/499, 60.72%) were in the age group 33–47, and 442/556, 79.50% of people lived in the most deprived areas (SIMD 1 or 2). Almost half of the patients (257/557, 46.14%) were seen by the ambulance service at their own home, or at a friend or relative’s house. Of those who were assessed elsewhere, 237/557 (42.55%) were assessed in public places such as supermarkets, on public transport (buses) or in the street.

**Table 1.** Demographics of the study population comprising 557 Tayside Substance Misuse Service patients who experienced a non-fatal overdose between 05/12/2017 and 12/05/2019.

Variable	Sample = *N*	*N*%
Age group (years)		
18–32	140	28.05
33–47	303	60.72
48–63	56	11.22
Gender	556	99.80
Male	434	78.06
Female	122	21.94
Location of patient documented by AS		
Police station/custody	47	8.44
Private property (own, friend’s or relative’s place)	257	46.14
Public place	237	42.55
NHS premise	16	2.87
Deprivation status		
SIMD 1 (most deprived)	229	41.19
SIMD 2	213	38.31
SIMD 3	55	9.89
SIMD 4	49	8.81
SIMD 5 (least deprived)	10	1.90

SIMD: Scottish Index of Multiple Deprivation; the numbers do not always add up to 557 due to missing variables.

The mean number (±standard deviation) of reported NFODs per person was 2.02 (±1.50), with a range of 1–13. The mean number (±standard deviation) of NFODs experienced by females and males was 1.60 (±1.50) and 2.14 (±2.05), respectively, (*p* = .006).

**Figure 1.**
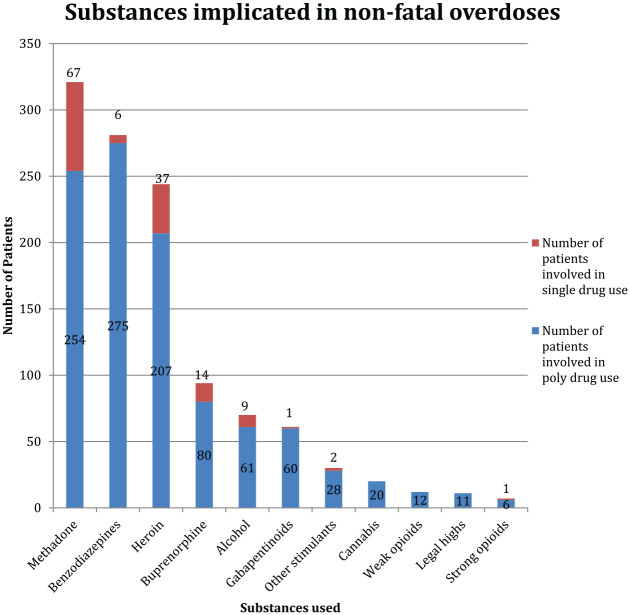
Overview of substances implicated in the 557 non-fatal overdoses, as reported by patients. The blue and yellow columns represent number of individuals who reported poly drug use and single drug use, respectively.

Figure 1 shows the drugs implicated in the NFODs. Methadone (321/557, 57.63%), benzodiazepines (281/557, 50.45%) and heroin (244/557, 43.81%) were the most frequently involved drugs. Polydrug use was common, and this was reported in 420/557 (75.40%) people. A combination of an opioid and a benzodiazepine was reported in around half of NFODs (275/557, 49.40%). In almost all NFOD cases where gabapentinoid consumption was reported, an opioid was also used (60/61, 98.40%): this included methadone (44/60) and heroin (28/60). Strong prescription opioids such as tramadol, hydromorphone, oxycodone, fentanyl and morphine and weak opioids such as co-codamol and codeine were implicated in 7 (1.26%) and 12 (2.15%) NFODs, respectively. Stimulants such as amphetamine and cocaine were used in 30 (5.39%) NFODs.

Of the patients where ORT prescription data were available, 400/439 (91.91%) were prescribed methadone and 213/439 (48.50%) buprenorphine. Amongst them, 174/439 (39.63%) patients were prescribed both methadone and buprenorphine, over the time period studied, but not at the same time. 69.50% (278/400) of the patients on methadone maintenance who suffered NFOD reported current methadone use, whereas only 32.90% (70/213) of patients on buprenorphine maintenance who suffered NFOD reported current buprenorphine use. This difference was significant (*p* < .001). NFODs, where consumption of non-prescribed ORT was reported, found 1/439 person not prescribed methadone but who overdosed on it, whilst buprenorphine was implicated in 24/429 people who were not prescribed this.

GCS was inconsistently recorded, and was complete in 363/557 patients. Amongst them 74 (20.38%) were classified as ‘severe’, 57 (15.70%) were ‘moderate’ and 232 (63.91%) were ‘mild’. Naloxone was administered in 362/557 (64.99%) of the NFODs by the ambulance service. In those people where GCS was recorded, allowing classification of NFOD severity, 55/74 (74.32%) patients with ‘severe’ NFOD, 41/57 (71.93%) patients with ‘moderate’ NFOD and 146/232 (62.93%) patients with ‘mild’ NFOD required naloxone. Table 2 shows, in those cases where GCS was used to assess overdose severity, NFODs taking place in a home or private accommodation were nearly five times more likely to be classified as ‘moderate’ or ‘severe’ (OR= 4.95, CI = 1.24, 24.38, *p* = .02) when adjusted for gender and age, compared to those that took place at NHS premises.

**Table 2.** Factors associated with moderate to severe Glasgow Coma Score in non-fatal overdoses.

Variable name	Odds ratio	95% confidence interval	*p*-Value
Age (per year)	0.99	0.96,1.02	.47
Gender			
Female	Reference		
Male	1.20	0.67, 2.17	.54
Location			
NHS premise	Reference		
Home/private accommodation	4.95	1.24, 24.38	**.02**
Public place	3.16	0.79, 15.54	.11
Police station/custody	3.89	0.82, 22.19	.09

Multi-variate logistic regression analysis adjusted for age and gender. Bold text: significant *p*-value.

Table 3 shows that males were two times more likely to be administered naloxone after an NFOD (OR = 1.94, CI = 1.11, 3.40, *p* < .02) than females, after adjustment for age, location and GCS. This could indicate that males are at a higher risk of a fatal overdose compared to females. Reported single or poly-drug was not significantly associated with other factors such as age, gender, GCS, location and naloxone use (see Supplementary Table).

**Table 3.** Factors associated with administration of naloxone.

Variable name	Odds ratio	95% confidence interval	*p*-Value
Age (per year)	0.97	0.94, 1.00	0.15
Gender			
Female	Reference		
Male	1.94	1.11, 3.40	**0.01**
Glasgow Coma Scale group			
Mild	Reference		
Moderate and severe	1.49	0.90, 2.52	0.12
Location			
NHS premise	Reference		
Home/private accommodation	0.49	0.07 2.26	0.40
Public place	0.46	0.06, 2.12	0.36
Police station/custody	1.29	0.15, 8.02	0.80

Multi-variate logistic regression analysis adjusted for age, location and GCS group. Bold text: significant *p*-value.

## Discussion

This study focuses on NFODs, as these are a major risk factor for future DRDs.^16,17^ Understanding the demographics of NFODs in people attending a specialist substance use service is important to develop robust care pathways to reduce NFODs and prevent fatal overdoses. Our key findings included that methadone, benzodiazepines and heroin were the drugs most frequently reported in NFODS, with the combination of a benzodiazepine and opioid being implicated in 49.4%. For those prescribed methadone, a higher proportion had their ORT involved as part of an NFOD (nearly 70%) than those prescribed buprenorphine (nearly 33%), with both commonly implicated in NFODs involving polydrug use. Amongst individuals reporting gabapentinoids consumption in their NFOD, 98.4% also reported taking an opioid. Males were at a higher risk of being administered naloxone and tended to have more NFODs. NFODs taking place at home or private accommodation were at a higher risk of being categorized with a moderate or severe GCS than those occurring in NHS premises.

Information on DRDs in Tayside indicates that 72% of the individuals were males, with an average age of 40.8^16^ – similar to our findings on NFODs. It is important to note that most of the DRDs reported by NHS Tayside had taken place at home (own or other’s) or supported accommodation, with only 9% taking place in public.^16^ We did find that moderate/severe impact on conscious level (as assessed by GCS) was more likely in NFODs at home or in private accommodation. In contrast to DRDs, 42.55% of the NFODs took place in public: this difference could mean that they had faster access to help by passersby, earlier access to emergency services or that the NFOD was less serious.^25,26^ A study observing the influence of environment on overdose found that family dynamics, inadequate emergency response and insufficient naloxone training resource increased risk of mortality.^27^ Training police officers and firefighters to administer intranasal naloxone is associated with decreased deaths in opioid overdose victims.^28,29^ Take home naloxone for those on ORT, with appropriate education on use, for individuals on ORT and/or their friends/family, has also been shown to be effective in reducing mortality.^30,31^ In people with chronic pain, a community-based intervention programme, called *Project Lazaurs*, was developed in partnership with physicians where naloxone (and training in its use) was provided to people with chronic pain prescribed long-term opioid therapy.^3^ In the first year of the programme, opioid-related deaths decreased by 50%.^3^ This suggests that educating people about the benefits of naloxone and co-prescribing could be a useful safety net in patients at risk of opioid OD, including those on ORT and those with chronic pain. Additionally, approaches that bring together the relevant multidisciplinary expertise through joint pain and addiction clinics need to be considered, for effective management to reduce harms and support people living with chronic pain and substance use issues.^32^

We found that males were more likely to be administered naloxone compared to females. The reasons for this are unclear, but may indicate that men had more severe NFODs, or alternatively could be an indicator of gender bias in perceived severity/need. Although our study did not find an association between gender and severity of overdose, previous research has found that being male was associated with increased mortality risk from drug overdose.^33,34^ It has been shown previously that in a study of drug-related deaths in the United States, when prescription painkillers were the primary cause of DRDs, gender difference in mortality narrowed, but when illegal drugs (such as heroin or non-prescription fentanyl) became the main cause, gender difference in mortality widened.^35^ Previous research has also found that emergency medical services were less likely to provide naloxone to women, older individuals and those without signs of illicit drug use during resuscitation attempts.^36,37^ With a rise in older, female and chronic pain patients who are at a high risk of prescription opioid use, first responders must consider opioid overdose among this patient population.^37^ It is important for clinicians to recognize gender-specific risk factors for NFODs as well as treatment initiation, continuation and treatment outcomes.^38^

Polydrug use was a clear issue in NFOD, and has also been found commonly in fatal OD. Etizolam was identified in toxicology by just over 62% of DRDs in Tayside in 2019, with heroin and methadone being the second and third common, respectively.^16^ There has also been a substantial increase in gabapentinoid prescribing in combination with opioids and benzodiazepines that has led to increased prevalence of gabapentinoids amongst DRDs.^9^ Additionally, there is accumulating evidence of the potential risk of gabapentinoid abuse amongst individuals with substance use problems.^11^ Drug-related deaths in Scotland 2020 report showed that, since 2015, there have been large increases in number of DRDs where ‘street’ benzodiazepines, methadone, heroin/morphine, gabapentinoids and cocaine were implicated.^15^ In contrast, strong and weak prescription opioids were only implicated in 3.41% of the NFODs in our study. The current trend in opioid use has seen a decrease in mortality associated with prescription opioids due to national initiatives of reducing opioid prescriptions.^39–41^ However, there has been a significant increase in overdose deaths from synthetic opioids and heroin.^39–41^ Our study has shown the high prevalence of opioids, benzodiazepine and gabapentinoid in NFODs. Further research is required on interventions to ensure safe and appropriate use of gabapentinoids and benzodiazepines.

Opioids used in substitution therapy, such as methadone and buprenorphine, also have a potential for overdose and drug diversion.^42^ There is a strong body of evidence on the benefits of methadone in treatment of opioid addiction.^43^ Apart from lowering risks of death, it lowers crime involvement and helps patients function better.^43^ Risk of opioid overdose caused by these medications is at highest during induction, and can be increased with the co-consumption of other sedative drugs such as benzodiazepines and alcohol.^44^ Indeed, we found that NFOD with methadone or buprenorphine in isolation was not common, as these drugs were usually taken in combination with another substance. We did show a disparity between patients taking methadone in NFOD, and those recorded as being prescribed methadone. A number of reasons for this could include that ambulance service staff do not enquire about ORT at the time of NFOD; or that there is medication non-adherence, which is a known risk factor for overdose.^45–49^ Currently, ambulance service personnel are only able to access GP prescribing data whilst off site attending callouts, but not community-based ORT prescribing by the local substance misuse service: future improvement to care pathways could include immediate access to ORT prescribing for ambulance service. Our study has shown the overdose potential of ORT and the prevalence of co-consumption of other sedative drugs, resulting in NFOD in patients attending SSMS. This study found that methadone was more likely to be implicated in NFODs than buprenorphine, and this is supported by a cohort study which showed that ORT with buprenorphine is associated with reduced mortality, largely due to decreased mortality in the initial month.^47^ The potential for buprenorphine diversion shown in our study is supported by a systemic review that found frequently cited reasons for non-prescription use of buprenorphine amongst individuals with opioid use disorder included therapeutic use to manage opioid withdrawal symptoms or achieve/maintain abstinence from opioids with a smaller percentage of individuals reporting reasons related to abuse.^50,51^

This study has a number of limitations. First, the regional ambulance service dataset did not include official toxicology reports and the drugs were identified by self-report by patients/carers/situation (drug packaging etc). Toxicology testing is not routinely carried out at the time of NFOD. Reporting of drugs could have been influenced by questions asked by the ambulance service staff, individual recall and accuracy of self-report, although there is some evidence that self-report may have additional benefit over toxicology in some situations.^52^ Agreement between urine toxicology and self-report, in patients admitted to hospital, has been shown to be generally high, but lower for opioids, benzodiazepines and heroin.^53^ Indeed, this may explain the lack of reporting of prescription opioids. Ideally, therefore, self-report should be combined with rapidly available toxicology, when considering service improvement approaches.

This study focused on a group of patients attending an SSMS, which limits the conclusions about risk factors relevant to general population. Future work is needed to look at the entire population along with co-morbidities (such as chronic pain and depression) and prescribed medications such as gabapentinoids and benzodiazepines to identify additional risk factors and reduce NFODs and fatal overdoses through timely interventions.

## Conclusion

Analysis of the regional ambulance service dataset of people with substance use issues who have been involved in an NFOD has added to understanding of a patient population that is at particularly high risk of OD (both fatal and non-fatal). This can be used to inform future service delivery and clinical management in order to reduce risk where possible. This study introduces a potentially useful approach to classification of NFOD severity: by GCS score and administration of naloxone. By classifying severity, we can identify individuals at high risk, and most in need of urgent intervention to prevent fatalities.

## Supplemental Material

sj-docx-1-bjp-10.1177_20494637221095447 – Supplemental material for Characteristics of non-fatal overdoses and associated risk factors in patients attending a specialist community-based substance misuse service Click here for additional data file. Supplemental material, sj-docx-1-bjp-10.1177_20494637221095447 for Characteristics of non-fatal overdoses and associated risk factors in patients attending a specialist community-based substance misuse service by Riya Ghose, Fiona Cowden, Abirami Veluchamy, Blair H Smith and Lesley A Colvin in British Journal of Pain

sj-docx-2-bjp-10.1177_20494637221095447 – Supplemental material for Characteristics of non-fatal overdoses and associated risk factors in patients attending a specialist community-based substance misuse service Click here for additional data file. Supplemental material, sj-docx-2-bjp-10.1177_20494637221095447 for Characteristics of non-fatal overdoses and associated risk factors in patients attending a specialist community-based substance misuse service by Riya Ghose, Fiona Cowden, Abirami Veluchamy, Blair H Smith and Lesley A Colvin in British Journal of Pain
