# Supplementary material for: Characteristics of non-fatal overdoses and associated risk factors in patients attending a specialist community-based substance misuse service
Source: Br J Pain. 2022 May 24;16(4):458–66. doi: 10.1177/20494637221095447 (PMC9411761; doi:10.1177/20494637221095447)
Supplement: sj-docx-1-bjp-10.1177_20494637221095447 – Supplemental material for Characteristics of non-fatal overdoses and associated risk factors in patients attending a specialist community-based substance misuse service [file sj-docx-1-bjp-10.1177_20494637221095447.docx]

***British Journal of Pain* Declarations Form**

**A declarations form must be submitted for ALL types of manuscript. *BJP* operates a double-blind peer review process, so please do not include names of any authors or institutions in this form as it will be seen by peer-reviewers.**

The [***BJP* declaration policy document**](http://journals.sagepub.com/pb-assets/BJP%20declaration%20policy%20document.pdf) should be read before filling in this form. This provides options for many of the declarations below.

**Title of Manuscript:** Characteristics of non-fatal overdoses, and associated risk factors in patients attending a specialist community-based substance misuse service

# Declaration of Conflicting Interest: LAC was a member of the MHRA Expert Working Group on Opioids; LAC, BHS were members of the Scottish Government Short Life Working Group On Prescription Medicine Dependence And Withdrawal.

**Funding:** Medical Research Scotland (VAC-1437-2019) supported this work

**Ethical approval** *(include full name of committee approving the research and if available mention reference number of that approval)***:** Caldicott approval (SMED Caldicott Guardian Approval 19/25) was obtained from the University of Dundee Caldicott Guardian. After discussion with the University of Dundee School of Medicine Ethics Committee, it was clarified that ethical approval was not required.

**Informed Consent:** Not Applicable.

**Trial Registration** *(where applicable)***:** Not Applicable

**Guarantor:** *LAC

**Contributorship:**  RG analysed the data and drafted manuscript. AV analysed the data and edited the manuscript. LAC, BHS and FC edited the manuscript. LAC, BHS, AV, RG and FC designed the project. All authors approved the final manuscript.

**Acknowledgements:** Medical Research Scotland (VAC-1437-2019) supported this work.

NOTE: Please do not leave any blank spaces. Appropriate wording can be found in the [***BJP* declaration policy document**](http://journals.sagepub.com/pb-assets/BJP%20declaration%20policy%20document.pdf)

------------------------------------------------------------------------------------------------------------------------

* The guarantor is the person willing to take full responsibility for the article, including for the accuracy and appropriateness of the reference list. This will often be the most senior member of the research group and is commonly also the author for correspondence. **Please use initials only:** *BJP* operates a double-blind peer review process so full names of authors should not be listed on this form.
