# Supplementary material for: Characteristics of non-fatal overdoses and associated risk factors in patients attending a specialist community-based substance misuse service
Source: Br J Pain. 2022 May 24;16(4):458–66. doi: 10.1177/20494637221095447 (PMC9411761; doi:10.1177/20494637221095447)
Supplement: sj-docx-2-bjp-10.1177_20494637221095447 – Supplemental material for Characteristics of non-fatal overdoses and associated risk factors in patients attending a specialist community-based substance misuse service [file sj-docx-2-bjp-10.1177_20494637221095447.docx]

Supplementary Table

**Table 1: Factors associated with poly drug use**

| Variable names | Odds Ratio (OR) | 95% confidence interval (CI) | p-value |
| --- | --- | --- | --- |
| Age | 0.98 | 0.94, 1.01 | 0.20 |
| Gender   - *Female* - *Male* | Reference  0.86 | 0.42, 1.67 | 0.67 |
| GCS Group   - *Mild* - *Moderate and Severe* | Reference  1.15 | 0.65, 2.04 | 0.63 |
| Location   - *NHS premise* - *Home/Private accommodation* - *Public place* - *Police station/ custody* | Reference  0.43  0.35  0.95 | 0.02, 2.56  0.02, 2.06  0.04, 8.95 | 0.44  0.34  0.97 |
| SIMD   - *SIMD 1 (most deprived)* - *SIMD 2 to 5* | Reference  1.09 | 0.62, 1.92 | 0.75 |
| Naloxone   - *Not required* - *Required* | Reference  0.82 | 0.45, 1.44 | 0.49 |

Note: SIMD: Scottish Index of Multiple Deprivation; GCS: Glasgow Comma Scale. Multi-variate logistic regression model adjusted for age, gender, GCS, location and naloxone.
